# Mehr als nur der Schmetterling – ein Leitfaden durch die Vielfalt des kutanen Lupus erythematodes: Pathogenese, Klinik, Therapie

**DOI:** 10.1007/s12326-021-00439-5

**Published:** 2021-04-09

**Authors:** P. M. Heil

**Affiliations:** Kollagenosen-Ambulanz, Universitätsklinik für Dermatologie, Medizinische Universität Wien, Währinger Gürtel 18–20, 1090 Wien, Österreich

**Keywords:** Systemischer Lupus erythematodes, Autoimmunität, Kollagenose, Immunsuppression, Interphasendermatitis, Systemic lupus erythematosus, Autoimmunity, Connective tissue disease, Immunosuppression, Inteface dermatitis

## Abstract

Die vielen klinischen Varianten des kutanen Lupus erythematodes (CLE) können solitär oder im Rahmen eines systemischen Lupus erythematodes (SLE) auftreten, auf dessen Vorkommen regelmäßig gescreent werden muss. Neben dem weiblichen Geschlecht und genetischen Faktoren stellen Sonnenexposition, Rauchen und manche Medikamente Risikofaktoren dar. Die wichtigsten CLE-Formen sind der akut-kutane LE (z. B. Schmetterlingserythem, generalisiert makulopapulös, enoral), der subakut-kutane LE (z. B. anuläre Form) und der chronisch-kutane LE (z. B. vernarbend diskoide Läsionen, Pannikulitis, Chilblain-LE). Die Diagnose beruht vor allem auf der Klinik und der Histopathologie, hinzu kommen autoimmunserologische Befunde und die direkte Immunfluoreszenz. Milde CLE-Formen können lokal therapiert werden. Reicht dies nicht aus, ist neben einem Steroidstoß Hydroxychloroquin die Systemtherapie der Wahl. Erweiterte therapeutische Optionen stellen Methotrexat, Retinoide, Dapson, Mycophenolat Mofetil, Azathioprin, Thalidomid, Belimumab und Rituximab dar. Alle CLE-Therapien sind off-label. Eine Aktualisierung der Impfungen sollte nach Möglichkeit vor Beginn einer Immunsuppression stattfinden. Zur Objektivierung des therapeutischen Ansprechens eines CLE empfiehlt sich das regelmäßige Scoring mittels RCLASI (Revised CLE Disease Area and Severity Index). Präventiv ist Sonnenschutz (Cremen, Kleidung, Reiseziele) von höchster Wichtigkeit, da Sonnenexposition Schübe provozieren kann. Ein LE stellt keine Kontraindikation gegen eine Schwangerschaft (SS) dar, jedoch sollte diese nicht in einem Schub eintreten, da dies das Risiko für Fetus und Mutter erhöht. Therapeutisch kommen während einer SS v. a. Steroide, Hydroxychloroquin, Dapson und Azathioprin in Betracht.

## Abstract (en)

The plethora of manifestations of cutaneous lupus erythematosus (CLE) can occur with or without concomitant systemic lupus erythematosus (SLE) for which regular screening is mandatory. Female sex, genetic predisposition, sun exposure, smoking and some medications serve as risk factors for LE. The most important CLE forms are the acute-cutaneous LE (e.g., butterfly rash, generalized macular–papular rash, enoral), the subacute-cutaneous LE (e.g., annular) and the chronic-cutaneous LE (e.g., scaring discoid lesions, panniculitis, chilblain-LE). Diagnosis is primarily based upon the clinical picture and its histopathology. Results of autoimmune serology and direct immunofluorescence can be helpful. For mild forms of CLE local therapy is often sufficient. If not, hydroxychloroquine is the first choice of systemic therapy in addition to transient systemic steroids. For recalcitrant cases additional therapy with methotrexate, retinoids, dapsone, mycophenolate mofetil, azathioprine, thalidomide, belimumab and rituximab may be used. All CLE therapies are off-label. An update of conventional vaccinations is advisable, if possible, to be performed before starting immunosuppressive therapies. To objectively verify the therapeutic response, the periodic scoring using the RCLASI (Revised CLE Disease Area and Severity Index) is recommended. Preventive sun protection (cream, clothing, travel destinations) is of pivotal importance, since intense UV-exposure can provoke disease flares. LE is no contraindication against a pregnancy. However, pregnancy should not start during a disease flare since it increases the risk for adverse outcomes for the fetus and mother. Steroids, hydroxychloroquine, dapsone and azathioprine serve as suitable therapies during pregnancy.

Der Lupus erythematodes (LE) ist eine entzündliche Autoimmunerkrankung, bei der einerseits ausschließlich die Haut in unterschiedlicher Art und Weise betroffen sein kann (*kutaner LE, CLE*), andererseits aber auch andere immunserologische (z. B. Autoantikörper) oder klinische Befunde abseits der Haut (z. B. Serositis, Nephritis etc.) positiv sein können: Diese Konstellation nennt man einen *systemischen Lupus erythematodes *(*SLE*, Klassifikationskriterien s. unten). Die Inzidenz des CLE liegt bei ca. 4/100.000 [1]. 75 % der SLE-Patienten entwickeln im Laufe ihrer Erkrankung eine der CLE-Formen [2].

## Pathogenese

Die exakte pathogenetische Kette des LE ist nicht im Detail geklärt, wohl aber sind wichtige Puzzlesteine bekannt, die wesentlich sind, um das autoaggressive Verhalten des Immunsystems in Gang zu setzen.

Das entzündliche Infiltrat des CLE ist dominiert von T‑Zellen (CD3+ Infiltrat), insbesondere von zytotoxischen T‑Zellen [3, 4], und attackiert die dermoepidermale Junktionszone. Einerseits durch diese entzündliche Attacke, andererseits auch durch exogene Einflüsse (starke UV-Exposition) kommt es zur Schädigung von Keratinozyten und Freilegung sonst intrazellulär gelegener Antigene, die nun erkannt und attackieret werden können [5]**. **Nach Sonnenexposition und Aktivierung der autoimmunen Reaktion kommt es typischerweise erst 1–2 Wochen später zu einer Verschlechterung bestehender oder Auftreten neuer CLE-Läsionen, da die oben geschilderte orchestrierte Immunreaktion Zeit benötigt, um voll in Gang zu kommen.

Hinzu kommt die Funktion von B‑Zellen. Zum einen sind diese Antigen-Präsentatoren („antigen-presenting cells“, APCs) für T‑Zellen, zum anderen sind sie Quelle der Plasmazellen, welche Autoantikörper produzieren, deren Zielantigene nach Zellschaden (s. oben) offenliegen. Dazu kommt, dass Keratinozyten nicht nur Ziel, sondern auch Akteur der Entzündungsreaktion sein können, indem sie Typ-I- und Typ-III-Interferone (IFN κ und IFNλ) sowie Interferon-abhängige Botenstoffe (z. B. CXCL10) produzieren [6–9]. Letzteres attrahiert CXCR3-positive T‑Zellen, was zur Nekroptose von Keratinozyten führt [10, 11].

Keratinozyten sind nicht nur Ziel, sondern können auch Akteur der Entzündungsreaktion sein

Wenngleich der LE als eine Erkrankung angesehen wird, dessen Pathogenese prototypisch für das erworbene Immunsystem (zytotoxisches T‑Zell-Infiltrat, Autoantikörper) ist, ist das angeborene Immunsystem jedoch ebenso involviert. So können zirkulierende Immunkomplexe (z. B. bestehend aus Autoantikörpern und RNA oder DNA) von plasmazytoid-dendritischen Zellen (CD123+HLADR+BDCA-2+) aufgenommen werden und mittels Aktivierung der endosomalen Rezeptoren TLR7 und TLR9 die Produktion von Typ-I-Interferon auslösen [12]. Der LE wird als prototypisch für eine Erkrankung mit Interferon-Signatur angesehen.

Ein weiteres interessantes Phänomen des angeborenen Immunsystems in der Pathogenese des SLE sind die „neutrophil extracellular traps“ (NETs), welche nicht nur beim SLE, sondern auch beim CLE beschrieben wurden [13]. NETs dienen eigentlich der Abwehr von Pathogenen (z. B. Bakterien), können jedoch auch DNA binden und dadurch Autoimmunität propagieren.

Die höhere Prävalenz von Frauen gegenüber Männern sowohl beim SLE [14] als auch beim CLE [15] ist lange bekannt, *Östrogene *spielen hier die wesentliche Rolle [16]. In den letzten Jahren konnten weitere, für einen LE prädisponierende Faktoren eruiert werden.

Es wurde eine Vielzahl von *Polymorphismen und genetischen Assoziationen* beschrieben [17], bis dato sind jedoch nur wenige monogenetische Varianten des CLE bekannt (TREX-1-Mutationen in TREX‑1, SAMHD1 sowie STING; [18–20]).

*Rauchen* ist mittlerweile als Risikofaktor für schwerere Verläufe des CLE etabliert [21].

Ebenso können Medikamente einen Lupus erythematodes provozieren („drug-induced lupus“, DIL; [22–24]). Zu den Auslösern gehören Procainamid, Hydralazin, Minozyklin, Terbinafin, Sulfasalazin, Carbamazepin, die Gruppe der Statine, der Kalziumkanal-Blocker und ACE-Hemmer und Checkpoint-Inhibitoren. Ebenso wurde in Fallberichten dokumentiert, dass ältere, chimär human-murine TNF-α-Blocker (Infliximab) bzw. modernere, humanisierte (Certolizumab) bzw. voll-human-rekombinante (Adalimumab) TNF-α-Blocker einen DIL hervorrufen können.

Rauchen ist als Risikofaktor für schwerere Verläufe des CLE etabliert

Zur Aktivität eines LE können zudem *bakterielle *[25] *und virale *[26]* Infektionen* beitragen; speziell auf letzteren liegt im Rahmen der COVID-19-Pandemie erneut ein spezielles Augenmerk [27].

**Figure 1:**
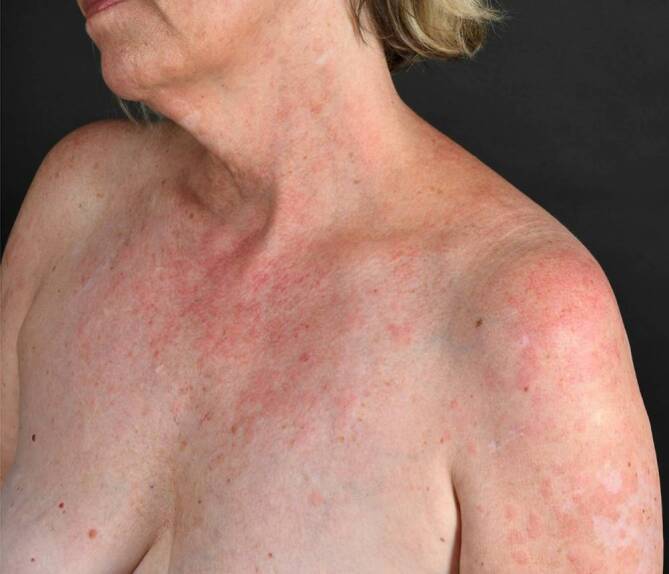

Nicht zuletzt sei darauf hingewiesen, dass *psychosoziale Stressoren* (z. B. Erkrankung oder Tod naher Bezugspersonen) zu LE-Schüben führen können ([28]; Abb. 1).

## Klinik

In der Abklärung eines Lupus erythematodes geht es aus dermatologischer Sicht immer um 2 Fragen:

1. Sind die Hautveränderungen, mit denen sich ein Patient vorstellt, ein CLE, und, falls ja, um welche CLE-Subform handelt es sich (s. unten)?
2. Besteht zusätzlich ein SLE?

In Bezug auf Hautveränderungen im Rahmen eines LE unterscheidet man spezifische Hautveränderungen, die nur beim LE vorkommen (CLE), von jenen Hautveränderungen, die unspezifisch sind und auch bei anderen Kollagenosen vorkommen können.

Die zwei wichtigsten Klassifikationen des CLE sind jene von Sontheimer [29] und Kuhn ([30]; Tab. 1).

**Table 1:** 

*Akut-cutaner LE (ACLE)*	*Schmetterlingserythem („butterfly-rash“)*
	Makulopapulöser ACLE
	TEN-like ACLE
	Mucosal ACLE
*Subakut-cutaner LE (SCLE)*	Anulär
	Papulosquamös
*Chronisch-cutaner LE (CCLE)*	Chronisch-diskoid (CDLE), limitiert
	Chronisch-diskoid (CDLE), disseminiert
	Lupus-Pannikulitis/Lupus profundus
	Hypertropher CDLE
	Chilblain-Lupus
	Mucosal CCLE
*Intermittierend-cutaner LE (ICLE)*	Lupus tumidus

Man unterscheidet beim CLE einen akut-cutanen LE (*ACLE*), von einem subakut-cutanen LE (*SCLE*) sowie einem chronisch-cutanen LE (*CCLE*). Diese Einteilung orientiert sich einerseits an der Aktualität bzw. der Chronizität des Geschehens, andererseits an der Morphe der Veränderungen.

Ein isolierter Hautbefall (ohne Beteiligung innerer Organe bzw. ohne positive Immunserologie) tritt vorzugsweise als CCLE auf; die höchste Wahrscheinlichkeit, parallel zur Haut auch an einem SLE zu leiden, hat der ACLE. Es sollte einem jedoch bewusst sein, dass selbst im Fall eines SLE Hautherde auftreten können, die von der Morphologie und vom Verlauf ein CCLE sind. Zudem können verschiedene CLE-Formen auch parallel auftreten (z. B. CCLE und ACLE).

**Figure 2:**
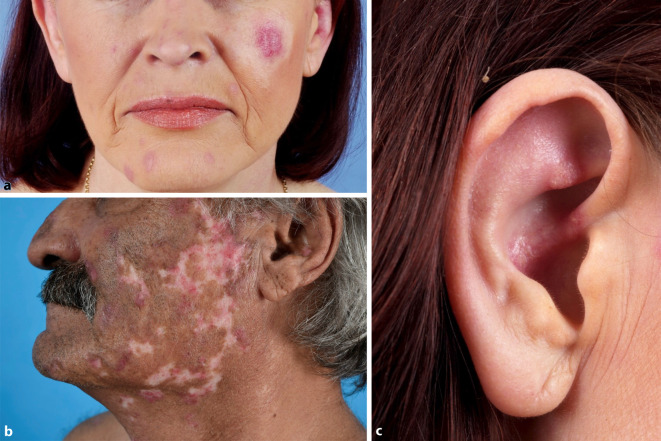

**Figure 3:**
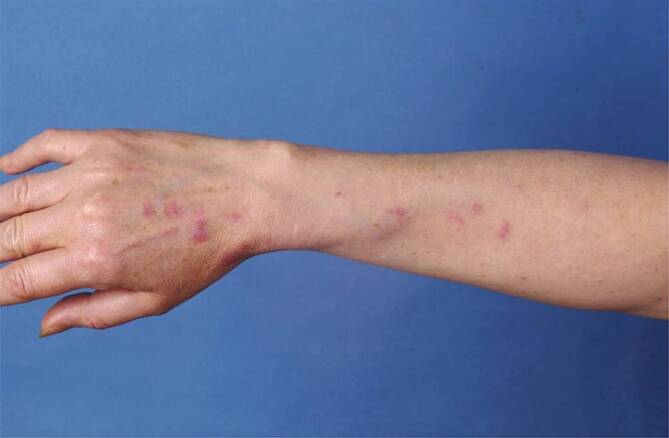

**Figure 4:**
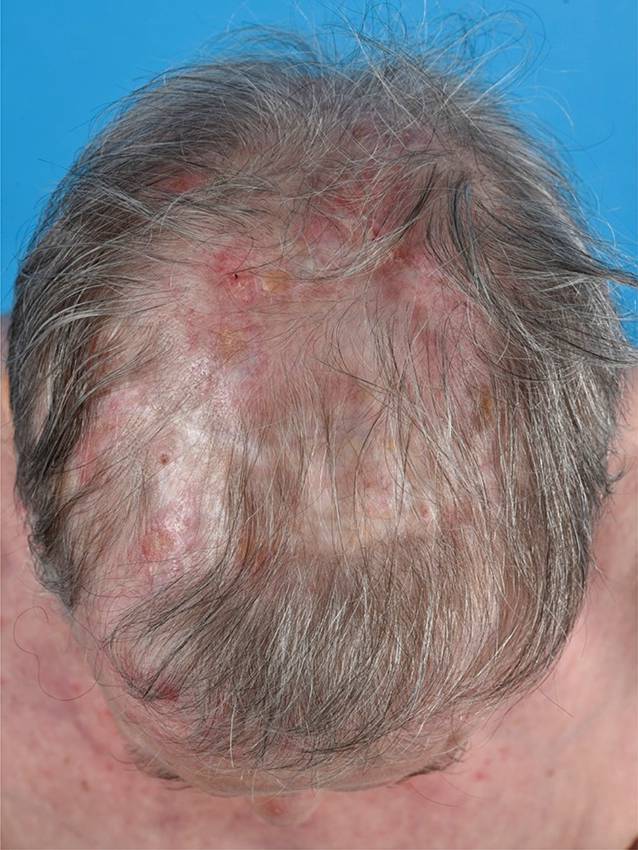

**Figure 5:**
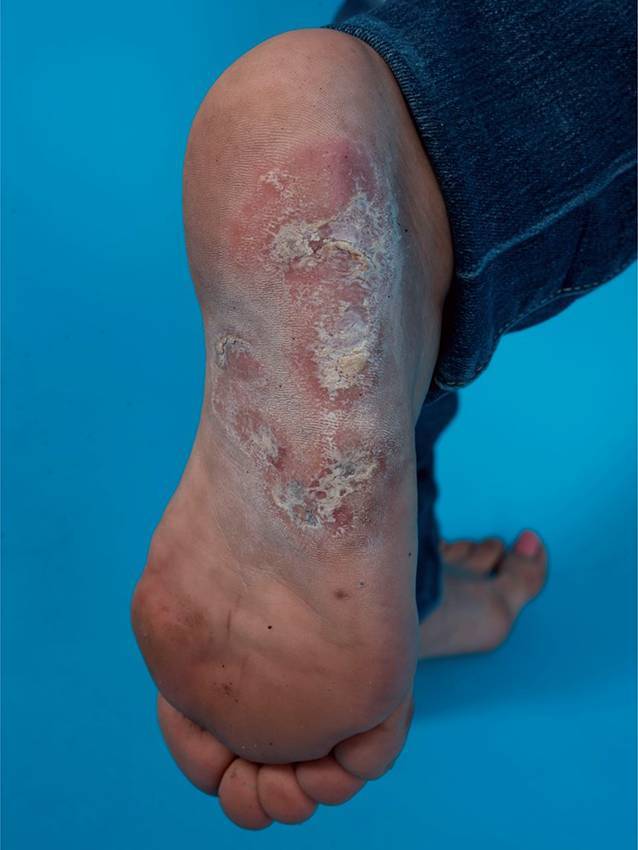

Die häufigste Variante des kutanen LE ist der *CCLE*. Eine Subform ist der chronisch-diskoide Lupus erythematodes (*CDLE*), welcher in der Regel durch einen isolierten Befall der Haut ohne Zeichen einer Systembeteiligung (Immunserologie, Organbeteiligung) gekennzeichnet ist und bevorzugt an sonnenexponierten Arealen auftritt, durchaus typisch ist ein Befall der Ohrmuschel. Ist nur der Kopf betroffen, spricht man vom *limitierten CDLE *(Abb. 2); ist auch der Rest des Körpers involviert, dann spricht man vom *disseminierten CDLE *(Abb. 3). Die Erkrankung verläuft chronisch und in Schüben, wobei die Schübe durch UV-Exposition getriggert werden können. Die ohne Therapie über Monate und Jahre an Größe zunehmenden Herde beginnen als schuppende erythematöse Plaques, die mit der Zeit zentral einsinken und vernarben bzw. zu einer vernarbenden Alopezie führen können (Abb. 4). Durch die Vernarbung kann es mit der Zeit sogar zu Mutilationen kommen. Eine Sonderform des CDLE ist der *hypertrophe CDLE *(Abb. 5), bei dem es durch eine ausgeprägte Hyperkeratose zu dicken Läsionen mit anhaftender Schuppung kommt.

**Figure 6:**
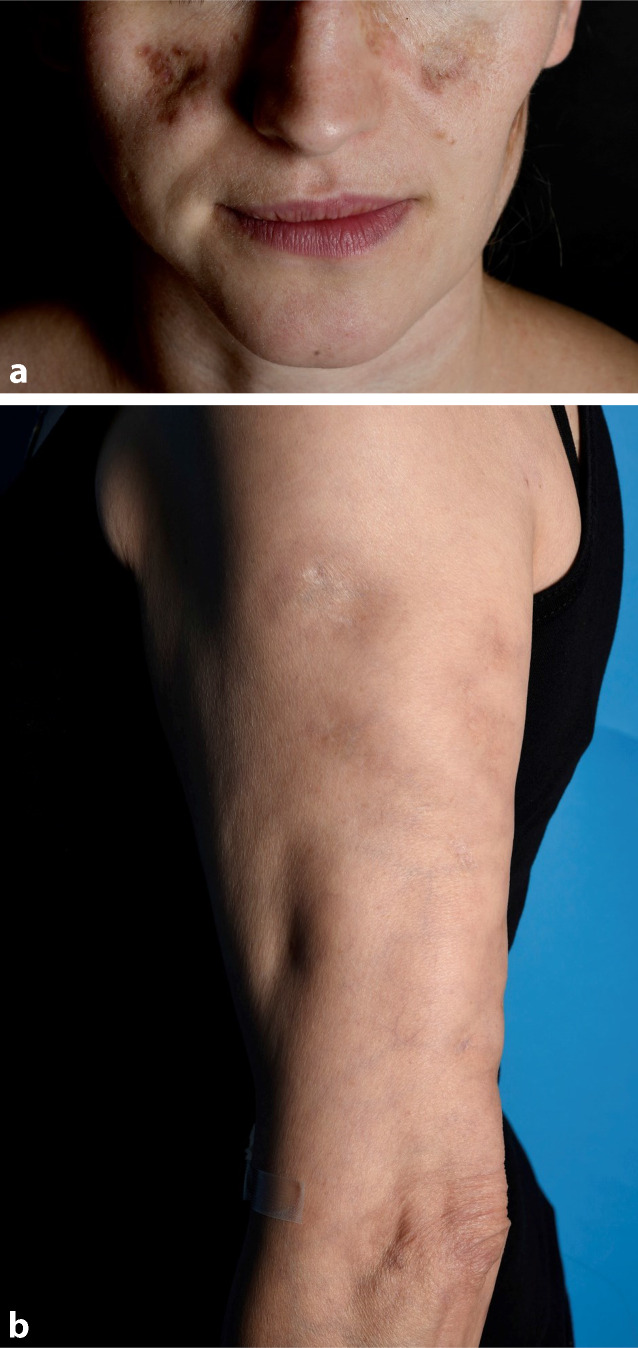

**Figure 7:**
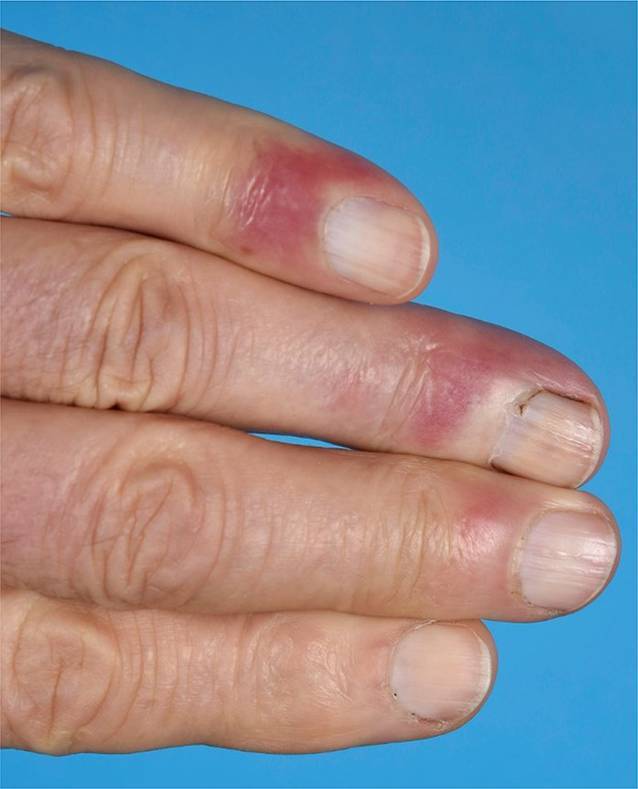

Andere Erscheinungsformen des CCLE an der Haut sind z. B. die *Lupus-Pannikulitis *(Abb. 6), der „*mucosal CCLE*“ oder der an Frostbeulen erinnernde und akral (Finger, Zehen, Nase, Ohren) auftretende *Chilblain-Lupus *(Abb. 7).

**Figure 8:**
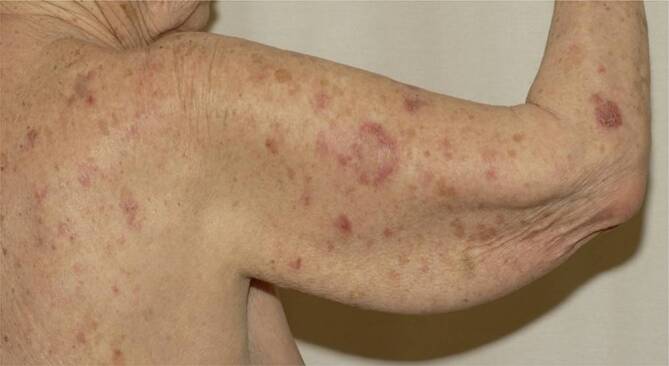

Der CCLE hat eine geringe Wahrscheinlichkeit (ca. 5 %), im weiteren Verlauf einen SLE zu entwickeln. Der SCLE ist überdurchschnittlich lichtempfindlich und ebenfalls durch schuppende, erythematöse, jedoch meist anulär angeordnete Plaques gekennzeichnet (*anulärer SCLE;* Abb. 8). Eine weitere Spielart des SCLE ist der *psoriasiforme SCLE.*

Die Herde des SCLE entwickeln sich rascher als beim CCLE, gelegentlich so rasch, dass die Schuppung als Zeichen der Chronizität fehlt. SCLE-Herde treten ebenfalls bevorzugt in den UV-exponierten Arealen auf. Auch die Histologie entspricht im Wesentlichen abgesehen von der Vernarbung der des CCLE. Der SCLE ist häufiger (ca. 25 %) mit einem SLE assoziiert als der CCLE. Eine deutliche Assoziation besteht auch mit Ro/SSA bzw. La/SSB-Autoantikörpern.

Die Erscheinungsformen des akut kutanen LE (ACLE) umfassen u. a. das weithin bekannte *Schmetterlingserythem*, welches lange Zeit unkritisch als Synonym für „Hautlupus“ und „SLE“ gesehen wurde. Wie wir mittlerweile wissen, gibt es viele unterschiedliche Arten des kutanen LE. Und: Auch ein Schmetterlingserythem kann solitär, ohne SLE, auftreten.

**Figure 9:**
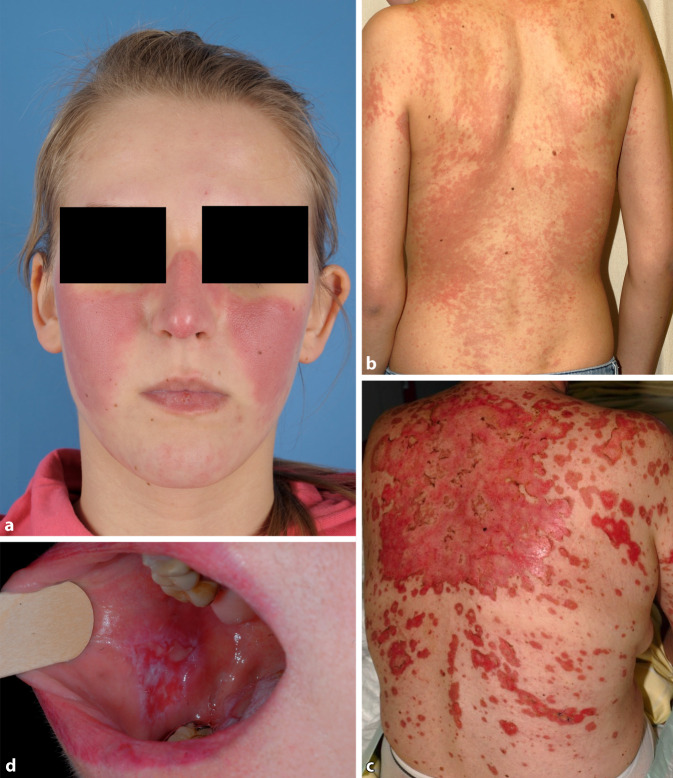

Beim Schmetterlingserythem (auch „butterfly rash“ genannt; Abb. 9a) handelt es sich um eine binnen weniger Wochen auftretende zentrofaziale Rötung im Bereich der Malarregion, welche auf die Nase übergreift und im Gegensatz zur Dermatomyositis die Augenlider in der Regel ausspart. Ödem und Schuppung können vorkommen.

Zum ACLE werden auch das generalisiert *makulopapulöse Exanthem* (Abb. 9b), die charakteristischen entzündlichen Plaques der Fingerstreckseiten (wobei typischerweise die Haut über den Gelenken ausgespart sind, auch dies im Gegensatz zur Dermatomyositis, wo diese typischerweise betroffen sind), die meist palmoplantar auftretende bullöse Form des ACLE, der an eine toxisch-epidermale Nekrolyse erinnernde „*TEN-like lupus*“ (Abb. 9c) sowie der „*mucosal ACLE*“ (Abb. 9d) gezählt. Die Formen des ACLE heilen in der Regel narbenlos ab. Der ACLE ist häufig Ausdruck eines SLE (ca. 75 %).

Eine Sonderform des CLE ist das sog. *Rowell-Syndrom*, bei dem sich zu einem ACLE oder CCLE anulär-multiformeartige Läsionen gesellen, die sehr an ein Erythema exsudativum multiforme erinnern.

Der* intermittierend kutane Lupus erythematodes *(*ICLE*; auch *Lupus tumidus *genannt) nimmt insofern eine Sonderstellung ein, als seine Klinik auch bei längerem Bestehen keine epidermale Komponente zeigt (entzündliches Infiltrat liegt tiefer), ganz besonders lichtempfindlich ist und ausgenommen selten in einen SLE übergeht. Die Klassifikation des ICLE ist seit Jahren Anlass zur Diskussion in Fachkreisen, die ihn entweder zum CCLE zuordnen [29] oder als eigene Kategorie führen [30].

Da Autoantikörper plazentagängig sind, können Neugeborene von Müttern, die an einem LE leiden, selbst daran erkrankt sein (*neonataler LE;* s. unten).

**Figure 10:**
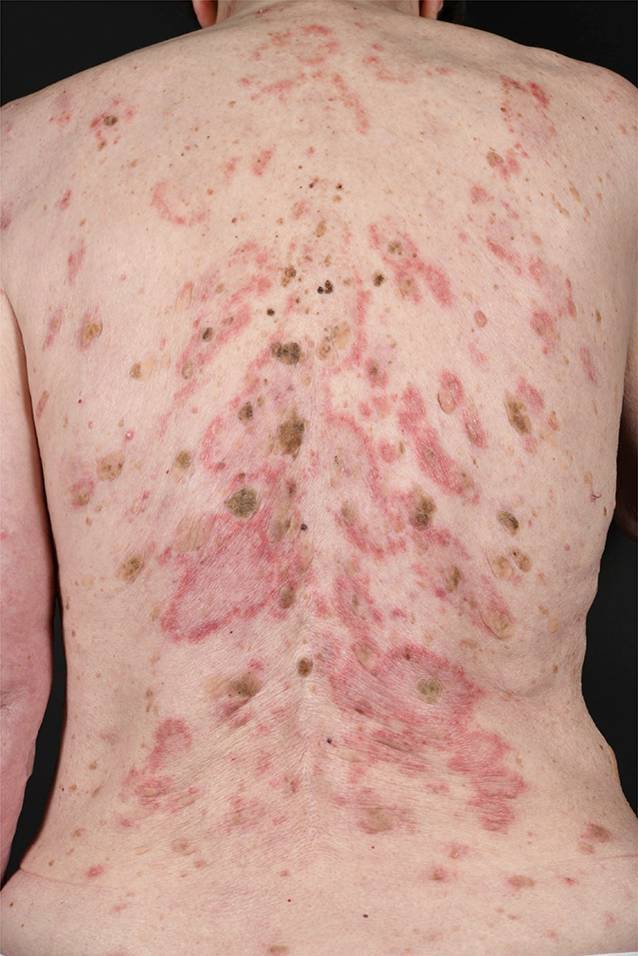

Unter dem Begriff „drug-induced lupus“ (DIL; Abb. 10; [22]) werden sowohl der medikamentös getriggerte CLE als auch das das „lupus-like syndrome“ (vorübergehendes SLE-ähnliches Bild, das meist nach Absetzen des kausalen Medikaments von alleine in Remission geht und selten Therapie benötigt) bzw. auch das Auftreten eines manifesten SLE (durch Demaskieren eine latenten SLE oder Hervorrufen eines Schubes eines bereits bekannten SLE) subsummiert. Das „lupus-like syndrome“ ist im Regelfall milder als ein idiopathischer SLE.

**Figure 11:**
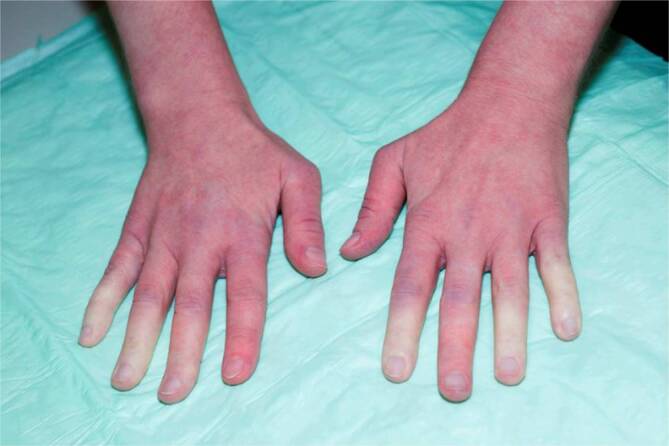

**Figure 12:**
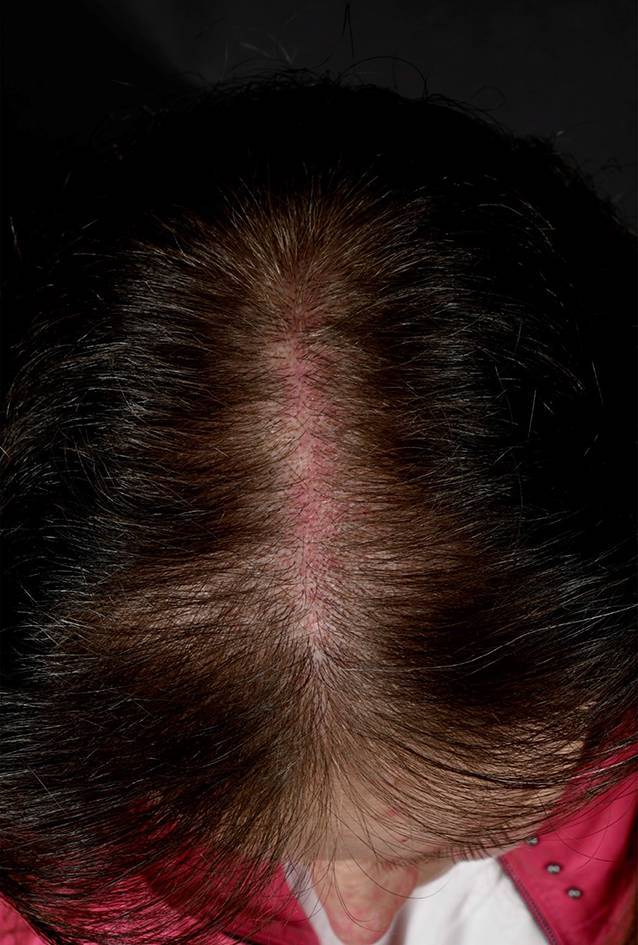

**Figure 13:**
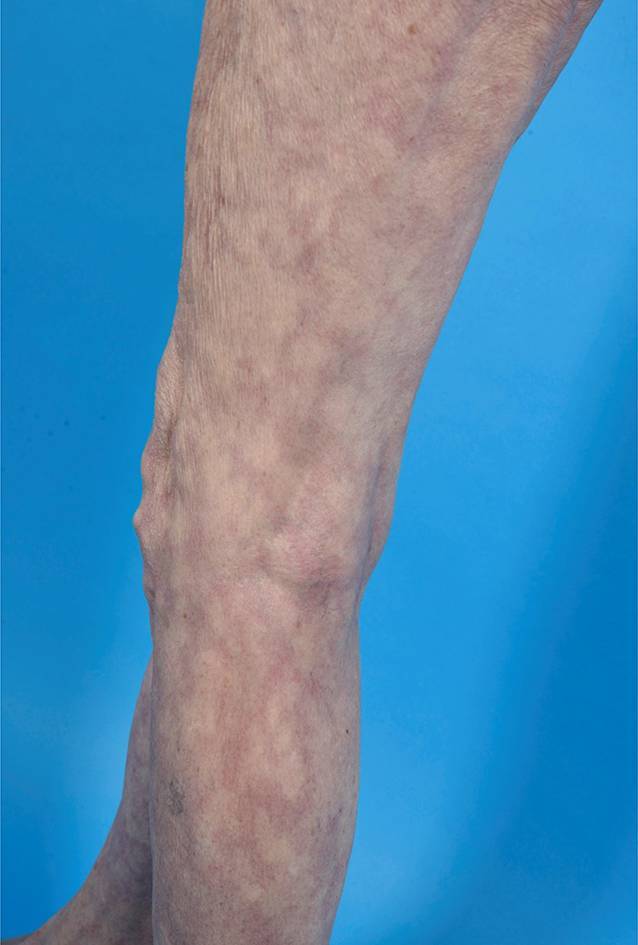

Zu den *unspezifischen kutanen Manifestationen*, die im Rahmen eines LE auftreten können, zählt das *Raynaud-Syndrom* (Abb. 11): Dieses ist definiert als spontan oder durch Triggerfaktoren wie Kälte oder Stress induzierte Spasmen der Fingerarterien mit triphasischer Hautreaktion: zuerst weiß (Anämie), dann blau (Hypoxie), dann rot (reaktive Hyperämie). Ebenso gehören in diese Kategorie die *Alopecia areata*, das *diffuse Effluvium* (Abb. 12), die *Livedo racemosa* (Abb. 13, durch Hypoxie ausgelöste rundlich livide Veränderungen, deren Kreise im Gegensatz zur physiologischen Livedo reticularis nicht geschlossen sind), Veränderungen der Nagelfalzgefäße (Kapillarmikroskopie) sowie eine *leukozytoklastische Vaskulitis.*

Die Organmanifestationen eines SLE sind mannigfaltig. Sie umfassen Nephritis, Arthritis, Pneumonitis, Myokarditis, Hepatitis und vieles mehr.

**Figure 14:**
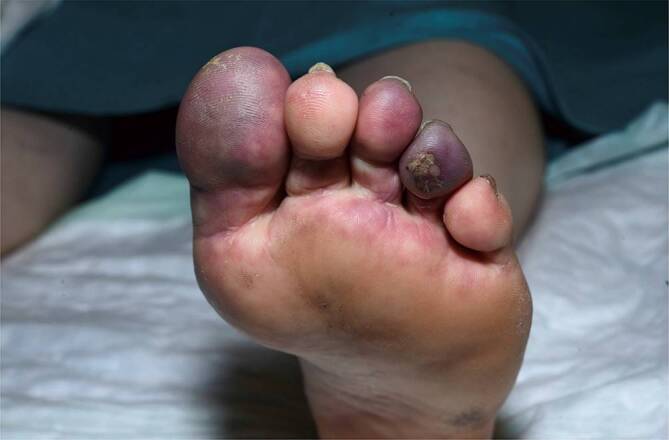

*Overlaps und assoziierte Syndrome:* Jede Kollagenose kann grundsätzlich mit anderen Kollagenosen klinisch und immunserologisch überlappen, insbesondere dem SLE. Dies bedeutet für die klinische Routine, dass die Anamnese und der klinische Blick auch Zeichen der Dermatomyositis, der systemischen Sklerose, des Sjögren-Syndroms und der rheumatoiden Arthritis erkennen sollten, um hier rechtzeitig eine weitergehende Abklärung und ggf. eine entsprechende Therapie einleiten zu können. Im weitesten Sinne kann man hier auch das *Antiphospholipid-Antikörper-Syndrom (APLAS)* nennen. Hier kann es durch Antiphospholipid-Autoantikörper (Cardiolipin, Beta-2-Glykoprotein I, Lupushemmstoff) neben Schwangerschaftskomplikationen (s. unten) zu einer Embolie im venösen oder arteriellen Gefäßbett kommen (Abb. 14).

## Diagnose

Die Diagnose eines CLE erfolgt anhand des geschilderten klinischen Bildes und der Ergebnisse von Hautbiopsien (Histologie bzw. direkte Immunfluoreszenz, DIF). Die Histologie der Formen des CLE ist entsprechend der heterogenen Klinik unterschiedlich; dazu kommt, dass die histologischen Befunde von der Dauer der Läsion abhängig und dadurch variabel sind. Ein typischer CCLE ist gekennzeichnet durch eine Differenzierungsstörung, ein „follicular plugging“, eine Verdickung der Balsamembranzone sowie ein perivaskuläres und periadnexielles Rundzellinfiltrat. Das typische Bild des SCLE besteht aus Interphasendermatitis, Aufsplitterung der Basalmembranzone und einem perivaskulären Rundzellinfiltrat, wohingegen der ACLE ein perivaskuläres Rundzellinfiltrat mit meist noch geringer Interphasendermatitis zeigt. Die LE-Pannikulitis ist primär lobulär und zeigt ein lymphozytäres Infiltrat, das zur Nekrose der Adipozyten führen kann, was sich klinisch in Eindellungen der Hautkontur ausdrückt. Beim bullösen ACLE besteht das entzündliche Infiltrat vor allem aus neutrophilen Granulozyten, der Spalt ist subepidermal, und häufig lassen sich Autoantikörper gegen Kollagen Typ VII nachweisen.

In der DIF, welche zur Vermeidung von falsch-positiven Befunden aus nichtlichtexponierter Haut entnommen werden sollte, zeigen sich granuläre Ablagerungen von vor allem IgG (aber auch IgM und C3) entlang der Basalmembranzone.

Die Diagnose des SLE erfolgte bis vor Kurzem hauptsächlich über die aus 1997 stammenden ACR-Kriterien des American College of Rheumatology [31]. Im Jahr 2019 wurden überarbeitete gemeinsame Kriterien des ACR und der europäischen Fachgesellschaft (EULAR) publiziert, die sich möglicherweise durchsetzen werden [32]. Auch diese sind wie die ACR-Kriterien eine Kombination aus klinischen und laborchemischen Kriterien, in denen die dermatologischen Manifestationen jedoch präziser (Alopezie, orale Ulzera, ACLE, SCLE, CDLE) abgebildet sind als bei den ACR-Kriterien.

Charakteristisch für den DIL sind Autoantikörper gegen Histone. Im Labor zeigt der DIL im Vergleich zum SLE seltener Zytopenien oder Erniedrigungen von Komplementfaktoren.

### Screening und Scoring.

Auch Patienten mit zunächst rein kutanem LE sollten regelmäßig auf das Vorliegen eines sich entwickelnden SLE untersucht werden, da dies mit oft nur geringen Symptomen wie z. B. chronischer Erschöpfung klinisch auffallen kann; dies trifft besonders auf die Nephritis zu, die im Gegensatz zu anderen Organbeteiligungen (z. B. Pleuritis) nahezu symptomlos ist. Zu solch einem Screening zählen neben einer gründlichen Anamnese Blutbefunde (BB, Diff-BB, Chemie, Gerinnung, ANA, AAK gegen ENA und dsDNA, Antiphospholipid AK, Lupushemmstoff) und Harnbefunde (Mikroalbumin-Kreatinin-Ratio, Protein-Kreatinin-Ratio, Harnsediment auf dysmorphe Erythrozyten).

Zur klinischen Quantifizierung der Ausdehnung eines CLE eignet sich der RCLASI (Revised Cutaneous Lupus Erythematosus Disease Area and Severity Index; [33]). Dieser beschreibt in allen anatomischen Regionen (inkl. Schleimhäute und Capillitium) das Ausmaß der aktiven Hautveränderungen (Erythem, Schuppung) sowie des bereits irreversibel eingetretenen Schadens (Vernarbung, Dyspigmentierung); man erhält am Ende zwei Ziffern, welche „activity“ und „damage“ benennen und zusammen den „total score“ ergeben. Ziel einer Therapie ist also, die Ziffern der Aktivität auf null zu drücken. Neben dem RCLASI empfiehlt es sich, eine genaue Fotodokumentation von Veränderungen durchzuführen, um im Verlauf Detailvergleiche durchführen zu können.

## Prävention und Therapie

Konsequentester Sonnenschutz stellt die zentrale Säule der Prävention dar. Die Information über den Zusammenhang zwischen UV-Exposition und Risiko eines LE-Schubs muss fixer Bestandteil der Patienteninformation sein. Präventionsmaßnahmen (Sunblocker, UV-Schutzkleidung, Wahl der Reiseziele) sollten regelmäßig besprochen werden.

Die Therapie des isoliert kutanen LE bzw. eines SLE, dessen einzig betroffenes Organ die Haut ist (*serokutaner SLE*), wird meist von Dermatologen durchgeführt. In Bezug auf die Therapie des CLE sei auch auf die gültige S2k-Leitlinie verwiesen [34].

Bei geringer Ausprägung des CLE ist eine Lokaltherapie (lokale Steroide [33] und Calcineurininhibitoren [35–37]) ausreichend; bei länger andauernder Lokaltherapie sollte Calcineurininhibitoren aufgrund des fehlenden atrophogenen Potenzials gegenüber Lokalsteroiden der Vorzug gegeben werden.

Neben einem systemischen Kortisonstoß (0,5–1,0 mg Prednisolon-Äquivalent/kg KG) als Akuttherapie ist die etablierteste steroidsparende Systemtherapie jene mit dem Antimalariamittel Hydroxychloroquin [38] in einer Dosierung von bis zu 6,5 mg/kgKG pro Tag (Tabletten zu je 200 mg), welche in der aktuellen S2k-Leitlinie auch als einzige steroidsparende First-line-Therapie geführt wird. Die wichtigsten Nebenwirkungen sind Ablagerungen in der Netzhaut sowie im Myokard, EKG-Veränderungen, Kopfschmerz, abdominelle Beschwerden (Inappetenz, Übelkeit, Diarrhoe) und Stimmungsschwankungen. Als seltene Nebenwirkung ist eine Typ- IV-Allergie beschrieben [39]. Regelmäßige ophthalmologische Kontrollen sind deshalb so wichtig, da Ablagerungen möglichst frühzeitig erkannt werden sollten, noch ehe subjektiv wahrnehmbare Sehstörungen auftreten. Dies ist umso wichtiger, als man trotz sofortiger Therapieunterbrechung auf die lange Halbwertszeit von Hydroxychloroquin von etwa 2 Monaten Rücksicht nehmen muss.

Rauchen sollte unbedingt beendet werden, da es nicht nur (s. oben) ein Risikofaktor für die Entstehung eines CLE ist, sondern zudem auch die therapeutische Wirksamkeit von Hydroxychloroquin schwächt [40].

Falls Hydroxychloroquin nach einigen Monaten zu keinem ausreichenden Erfolg führt, ist das nächste Immunsuppressivum der Wahl Methotrexat (Second-line-Therapie; [41]). Ist auch mit Methotrexat keine Remission zu erzielen oder besteht eine Kontraindikation, kommen die beiden anderen Second-line-Optionen Retinoide ([42]; insbesondere beim hypertrophen CDLE) und Dapson ([43]; insbesondere beim bullösen ACLE) zum Einsatz.

Weitere Therapeutika (alle Third-line-Therapien) stellen Mycophenolat-Mofetil [44], Thalidomid (strengste duale Kontrazeption; [45]) sowie Azathioprin (während Schwangerschaft einsetzbar; [46]) dar.

Das einzige derzeit für den SLE zugelassene Biologikum, Belimumab (Hemmer des B‑lymphozyte stimulators [BlyS] = B-Cell Activating Factor [BAFF]), hat in ersten Beobachtungen eine gute Wirkung auf kutane LE-Formen gezeigt [47]. In Einzelfällen können Immunglobuline (i.v. oder s.c.) oder Rituximab eine sinnvolle Option darstellen, insbesondere dann, wenn ein SLE im Hintergrund steht [48].

Die o. g. Reihung (First‑/Second‑/Third-line) ist als Empfehlung zu verstehen für den Fall, dass die Haut die einzige Endorganaktivität darstellt. Im klinischen Alltag kommt es regelmäßig vor, dass neue Aspekte, die zusätzlich zum CLE auftreten, eine andere Therapiegewichtung erfordern (z. B. MMF bei Nierenbeteiligung oder Lungenbeteiligung, Azathioprin bei Schwangerschaft, Dapson bei Neutrophilen-getragener Vaskulitis usw.).

Falls es die Akuität der Erkrankung zulässt, sollte noch vor Beginn einer Immunsuppression eine Aktualisierung der Impfungen durchgeführt werden, spätestens parallel zur begonnen Therapie, um das erhöhte Infektionsrisiko zu minimieren.

Aufgrund des Sonnenschutzverhaltens der Patienten sollte regelmäßig eine Kontrolle des Vitamin-D-Spiegels erfolgen und entsprechend suffizient und nachkontrolliert ein Mangel ausgeglichen werden.

Beim medikamentös induzierten Lupus erythematodes ist die wichtigste Maßnahme die Identifikation des kausalen Agens und dessen Absetzen. Sollte dies nicht ausreichen, ist therapeutisch gemäß dem idiopathischen LE vorzugehen.

### Medicolegaler Aspekt.

Wichtig ist zu betonen, dass keine der genannten Systemtherapien für die Therapie des CLE zugelassen ist und man sich als Dermatologe hier im off-label bewegt, weshalb eine besonders gründliche, auch schriftliche, Aufklärung erfolgen sollte, wofür substanzspezifische Aufklärungsreverse existieren.

## Lupus erythematodes und Schwangerschaft

Ein CLE, aber auch ein SLE, stellt keine grundsätzliche Kontraindikation gegen eine Schwangerschaft dar, jedoch sollte diese mit einer auf Risikoschwangerschaften spezialisierten Ambulanz geplant und von dieser begleitet werden. Therapeutisch stehen während der Schwangerschaft Steroide, Hydroxychloroquin, Azathioprin, Immunglobuline (i.v. oder s.c.) und Dapson zur Verfügung. Eine Nutzen-Risiko-Abwägung muss erfolgen.

Keinesfalls sollte eine Schwangerschaft während eines Krankheitsschubs stattfinden, da dies die Komplikationsrate für Mutter und Kind erhöht. Kommt es zu einer Schwangerschaft während eines LE-Schubs, sollte unverzüglich die Zuweisung an ein spezialisiertes Zentrum erfolgen.

Exemplarisch sei auf die folgenden beiden Problemkonstellationen hingewiesen: Das Antigen zu mütterlichen Ro/SSA- bzw. La/SSB-Autoantikörpern ist noch auf dem fetalen Reizleistungssystem exprimiert und führt bei ca. 2 % der Feten/Neugeborenen zum „cardiac neonatal lupus erythematosus“ (C-NLE). Das klinische Spektrum reicht von Herzrhythmusstörungen bis hin zum Tod (ca. 20 % der C‑NLE-Fälle). Etwa 70 % der C‑NLE-Fälle benötigen einen Schrittmacher [49–51]. Screening-Methode der Wahl ist das fetale EKG (erweiterte Methode des Tocogramms). Die therapeutischen Optionen reichen von plazentagängigen Steroiden bis hin zur Apherese [52]. Ro/SSA- bzw. La/SSB-positive Schwangere sollten auch ohne Klinik präventiv Hydroxychloroquin erhalten [53].

Ein APLAS kann Aborte, Frühgeburten und (Prä‑)Eklampsie auslösen. Mütter mit positiven Antiphospholipid-Antikörpern sollten auch ohne stattgehabtes klinisches Ereignis präventiv 100 mg Acetylsalicylsäure (ASS) erhalten [53].

## Conclusio

Klinik und Histopathologie des kutanen Lupus erythematodes sind vielfältig, was im klinischen Alltag eine Herausforderung darstellen kann. Dies gilt auch für das therapeutische Armamentarium, das breit ist und an die individuelle Konstellation des Patienten angepasst werden muss. Ein Screening (Blut- und Harnbefunde) auf einen parallel auftretenden systemischen Lupus erythematodes ebenso wie auf Overlap-Syndrome sollte regelmäßig und standardisiert erfolgen. Ein Lupus erythematodes stellt keine Kontraindikation für eine Schwangerschaft dar, zumal ausreichend sichere und effektive Therapien existieren.
